# Supplementary material for: Inhibitor of CD147 Suppresses T Cell Activation and Recruitment in CVB3-Induced Acute Viral Myocarditis
Source: Viruses. 2023 May 10;15(5):1137. doi: 10.3390/v15051137 (PMC10221111; doi:10.3390/v15051137)
Supplement: Supplementary file 1 [file viruses-15-01137-s001.zip › viruses-2375145-supplementary.pdf]

Supplements

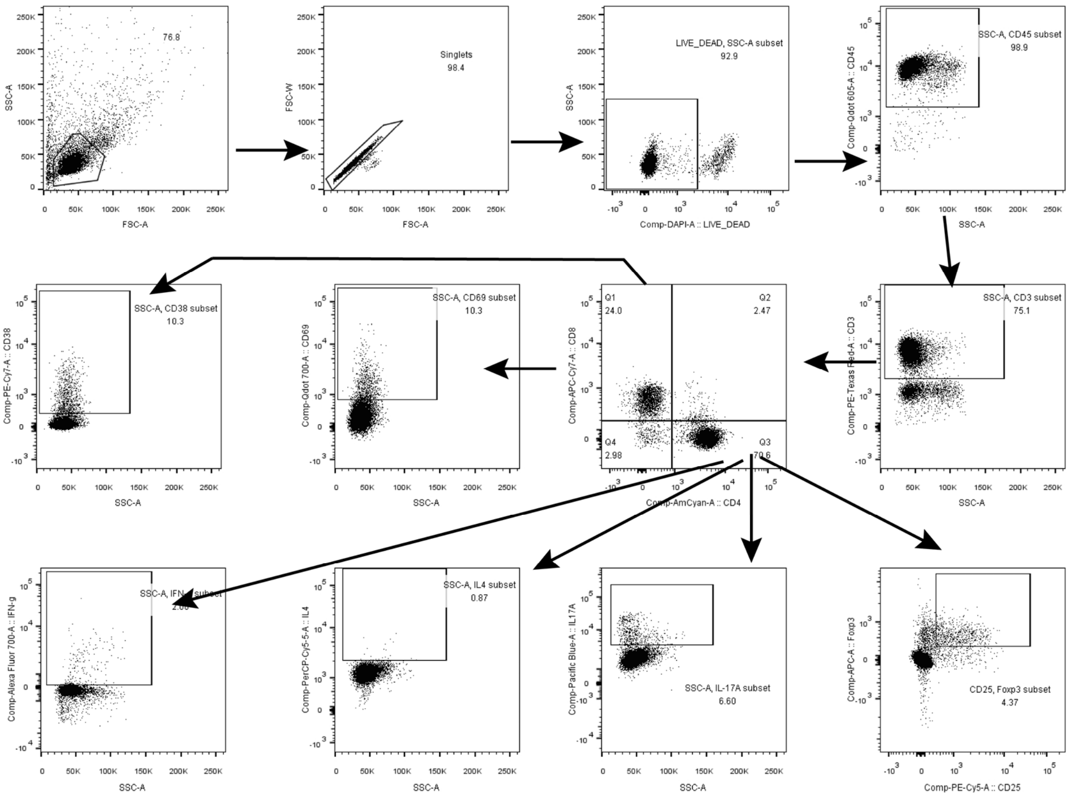

Supplementary Figure S1. Representative gating strategy for immune cells.

Supplementary Table S1. The sequence of primers for cytokines.

Cytokines	Sequence 5-3’	Annealing Temperature
IFN-γ Forward	TCAAGTGGCATAGATGTGGAAGAA	60°C
IFN-γ Reverse	TGGCTCTGCAGGATTTTCATG	
IL-17 Forward	CTCAAAGCTCAGCGTGTCCAAACA	60°C
IL-17 Reverse	TATCAGGGTCTTCATTGCGGTGGA	
IL-10 Forward	AGCCTTATCGGAAATGATCC	55°C
IL-10 Reverse	GGGAATTCAAATGCTCCTTG	
GAPDH Forward	AGGTCGGTGTGAACGGATTTG	60°C
GAPDH Reverse	TGTAGACCATGTAGTTGAGGTCA	
